# Whole-Genome and Expression Analyses of Bamboo Aquaporin Genes Reveal Their Functions Involved in Maintaining Diurnal Water Balance in Bamboo Shoots

**DOI:** 10.3390/cells7110195

**Published:** 2018-11-02

**Authors:** Huayu Sun, Sining Wang, Yongfeng Lou, Chenglei Zhu, Hansheng Zhao, Ying Li, Xueping Li, Zhimin Gao

**Affiliations:** 1 State Forestry Administration Key Open Laboratory on the Science and Technology of Bamboo and Rattan, Institute of Gene Science for Bamboo and Rattan Resources, International Centre for Bamboo and Rattan, Beijing 100102, China; huayu.sun@uconn.edu (H.S.); wangsining66@163.com (S.W.); zhuchenglei@icbr.ac.cn (C.Z.); zhaohansheng@icbr.ac.cn (H.Z.); liying105527@126.com (Y.L.); lxp@icbr.ac.cn (X.L.); 2 Department of Plant Science and Landscape Architecture, University of Connecticut, Storrs, CT 06269, USA; 3 Jiangxi Academy of Forestry, Nanchang 330013, China; louyf1983@163.com

**Keywords:** aquaporins, AQPs, bamboo, root pressure, fast growth

## Abstract

Water supply is essential for maintaining normal physiological function during the rapid growth of bamboo. Aquaporins (AQPs) play crucial roles in water transport for plant growth and development. Although 26 *PeAQP*s in bamboo have been reported, the aquaporin-led mechanism of maintaining diurnal water balance in bamboo shoots remains unclear. In this study, a total of 63 *PeAQP*s were identified, based on the updated genome of moso bamboo (*Phyllostachys edulis*), including 22 *PePIP*s, 20 *PeTIP*s, 17 *PeNIP*s, and 4 *PeSIP*s. All of the *PeAQP*s were differently expressed in 26 different tissues of moso bamboo, based on RNA sequencing (RNA-seq) data. The root pressure in shoots showed circadian rhythm changes, with positive values at night and negative values in the daytime. The quantitative real-time PCR (qRT-PCR) result showed that 25 *PeAQP*s were detected in the base part of the shoots, and most of them demonstrated diurnal rhythm changes. The expression levels of some *PeAQP*s were significantly correlated with the root pressure. Of the 86 sugar transport genes, 33 had positive co-expression relationships with 27 *PeAQP*s. Two root pressure-correlated *PeAQP*s, *PeTIP4;1* and *PeTIP4;2*, were confirmed to be highly expressed in the parenchyma and epidermal cells of bamboo culm, and in the epidermis, pith, and primary xylem of bamboo roots by in situ hybridization. The authors’ findings provide new insights and a possible aquaporin-led mechanism for bamboo fast growth.

## 1. Introduction

Bamboo plays a very important role in reducing soil erosion and enabling land restoration and water conservation [1]. Fast growth is one of the most prominent features of bamboo. As the most representative species, moso bamboo (*Phyllostachys edulis*) can grow up to more than 1 m in 24 h and reach more than 20 m over one-and-a-half months in spring [2]. As a clonal plant, bamboo establishes its extensive underground rhizome network for water and nutrient absorption and transportation. Numerous studies have shown that the fast growth of bamboo shoots is entirely dependent on a well-developed rhizome network, which can spread widely horizontally and connect the young culms with other mature bamboos [1,3,4]. Plants must adjust the supply of water through the root system to the demand for water by the shoots, to sustain transpiration water loss and carbon fixation [5]. A strong water potential gradient is a key factor for water transportation from mature to young bamboo [6]. Bamboo rapid growth rates and highly developed rhizome systems connecting culms underground suggest that bamboos may have different hydraulic architectures and water-use strategies, compared with dicotyledonous woody plants [7]. The supply of water is essential for maintaining normal physiological functions during the rapid growth of bamboo [8]. Moreover, both water potential gradient and root pressure are two essential factors for woody bamboo to maintain diurnal water balance and to drive fast growth [6,9].

Unlike trees, bamboos have a hollowed culm and they lack secondary growth, suggesting that the vascular conduits in bamboo xylem have to remain functional throughout their life [10]. In other words, bamboos have a strong ability to avoid cavitation that is caused by xylem embolism. Root pressure that is transported by highly developed rhizome systems may contribute to repairing xylem embolism at night [9], and it could be helpful for the growth and persistence of bamboo [11]. Moreover, root pressure is important for woody bamboos to maintain diurnal water balance [7]. All of those features and structural traits of bamboos may explain their rapid growth mechanism. Bamboo shoots lack branches and leaves, which is an appropriate model to investigate the diurnal variations of root pressure.

As water channel proteins, aquaporins (AQPs) can maintain water and hydraulic conductivity balance in plants [12]. AQP activity influences a range of physiological processes, such as phloem loading, xylem water exit, stomatal aperture, and gas exchange [13]. Large amounts of AQP homologous proteins have been found in plants, and they are divided into five major subfamilies: plasma membrane intrinsic proteins (PIPs), tonoplast intrinsic proteins (TIPs), nodulin 26-like intrinsic proteins (NIPs), small basic intrinsic proteins (SIPs), and uncharacterized X intrinsic proteins (XIPs) in most dicots [14,15,16,17]. However, XIPs are not found in monocots and certain dicots (Table S1), such as *Phyllostachys edulis* [8] and *Arabidopsis thaliana* [18]. With the first bamboo whole-genome sequencing draft completion [2], a total of 26 aquaporin genes were identified in the moso bamboo genome, including ten PIPs, six TIPs, eight NIPs, and two SIPs [8]. Those expression levels were monitored in roots, stems, leaves, and sheaths, as well as those in roots and leaves under drought, water, and NaCl stresses using quantitative real-time PCR (qRT-PCR) [8]. Although systematic expression analyses of the *PeAQP* family have been performed, actual functional identification was rare. Until now, only one aquaporin gene, *PeTIP4;1-1*, has been cloned and functionally analyzed [19]. Its expression level was elevated with the increasing height of the bamboo shoots, and significantly up-regulated in response to drought and salinity stresses in roots and leaves. Furthermore, overexpression of *PeTIP4;1-1* in *A. thaliana* showed enhanced tolerance under drought and salinity stresses. The functions of the other bamboo AQPs remain unknown.

In this study, the authors expanded the members of moso bamboo *AQP*s (*PeAQP*s) from 26 to 63, based on the updated annotation information from the Genome Atlas of Bamboo and Rattan (GABR) Project, using both Illumina (Illumina lnc, San Diego, CA) and PacBio (Pacific Biosciences of California Inc., Menlo Park, CA) sequencing platforms [20]. The phylogenetic relationships, gene structures, and conserved motifs of 63 *PeAQP*s were further analyzed, and their expression profilings in the rhizome bud, as well as the top, middle, and base parts of shoots with heights of 0.2, 1.5, 3, and 6.7 m, were generated. In addition, the diurnal variation of root pressure in ~6 m shoots was measured. A correlation between the root pressure and the expression levels of *PeAQP*s was analyzed. Furthermore, a co-expression network of *PeAQP*s and sugar transport genes was constructed. The expression of two *PeAQP*s that were positively and negatively correlated with root pressure was further analyzed by in situ hybridization. Finally, the relationship of the *PeAQP*s and root pressure with bamboo’s rapid growth were proposed and discussed.

## 2. Materials and Methods

### 2.1. Identification and Structural Analysis of *AQP* Proteins and Sugar Transporters in Moso Bamboo

Based on the updated data of moso bamboo (http://gigadb.org/dataset/100498, 1 October 2018) [20], the putative AQP genes in moso bamboo were re-identified completely, using ‘aquaporin’ and ’water channel’ as the keywords. For sequence identification and screening, the sequences lacked any of the conserved domains of the Asn–Pro–Ala (NPA) motifs, and the aromatic/arginine (ar/R) selectivity filter, which is indispensable in determining the transport specificity of the channel, was excluded after the comprehensive analyses [21,22,23,24].

The distribution of the introns and exons were analyzed, following the reference method [8]. The result was shown using the online software GSDS 2.0 (Center for Bioinformatics, Peking University, Beijing, China) (http://gsds.cbi.pku.edu.cn/). The subcellular localization of the PeAQPs was predicted with the online tool Plant-mPLoc (Institute of Image Processing & Pattern Recognition, Shanghai Jiaotong University, Shanghai, China) (http://www.csbio.sjtu.edu.cn/bioinf/plant-multi/). Transmembrane (TM) helices were predicted using the online tool TMpred (Bioinformatics Group, Koeln, Germany) (https://embnet.vital-it.ch/software/TMPRED_form.html).

Sugar transport genes were re-identified using the keywords of sugar transport, sucrose transporter, SWEET, monosaccharide transporter, hexose transporter, polyol transporter, myo-inositol transporter, plastidic glucose transporter, monosaccharide-sensing protein, sugar facilitator, and so on. The sequences of each sugar transport gene are listed in Table S2. Further analyses of proteins encoded by the sugar transport genes were performed by using methods similar to those for aquaporins.

### 2.2. Phylogenetic Analysis of AQPs

To investigate the phylogenetic relationship of aquaporins, a clustering analysis of AQPs in *P. edulis*, *Zea mays*, and *Oryza sativa* was constructed in MEGA6.0 (Research Center for Genomics and Bioinformatics, Tokyo Metropolitan University, Hachioji, Tokyo, Japan) using the following parameters: the neighbor-joining tree method, partial deletion, and bootstrap with 1000 replicates. The sequences of *ZmAQP*s and *OsAQP*s were downloaded from the MaizeGDB (Cold Spring Harbor Laboratory, Cold Spring Harbor, NY, USA) [25] and Ricedata (China Rice Data Center, China) (http://www.ricedata.cn/gene/) [26], based on published research [27,28]. In addition, the sequences of *PtAQP*s in *Populus trichocarpa* were downloaded to investigate the presence of XIP aquaporins in bamboo [14].

### 2.3. Tissue-Specific Expression Pattern Analysis of Bamboo AQP Genes

To investigate the expression of *PeAQP* genes in the different tissues and developmental stages of shoots, transcriptome data generated from 26 tissues of rhizome, roots, shoots, leaves, sheaths, and buds during different development stages were downloaded from the NCBI Short Read Archive (SRA) [20] and used for further gene expression analysis. The accession numbers in SRA were SRX2408703~SRX2408728, respectively. The expression levels were normalized by log_2_ and loaded into Matrix2png (Columbia Genome Center, Columbia University, NY, USA) (http://www.chibi.ubc.ca/matrix2png/) to generate a heat map.

### 2.4. Measurement of Root Pressure

Root pressure dynamics were monitored at the base of six different moso bamboo shoots at ~6 m height from 7:00 p.m. on 20 April 2017 to 7:00 p.m. on 22 April 2017. The measurement experiments were undertaken in a field site belonging to the Jiangxi Academy of Forestry (28°44′40″ N, 115°48′41″ E, 41 m), Nanchang, Jiangxi Province, which is a research partner of the authors’ institute. During the experiment, the selected bamboo shoots were crosscut at the base part, 10 cm above the ground. The shoot cross-section was polished with a fresh razor blade, then covered with a plastic tube made by a Le buckle cup filled with distilled water. The cup was connected to a digital pressure transducer (PX26-100DV, Omega Engineering, Stamford, CT, USA) linked to a data logger (CR1000, Campbell Scientific, Logan, UT, USA); the pressure data were collected every 10 s according to the method described in previous studies [7,9,11].

### 2.5. Correlations and Network Analyses

The correlation between the gene expression level of the *PeAQP*s and the root pressure in the base part of the bamboo shoots was constructed by calculating pairwise Pearson correlation coefficients (PCC) based on the transcriptome data of *PeAQP*s across the 26 moso bamboo samples, which were then loaded into Java TreeView 1.1.6r4 (Division of Environmental and Evolutionary Biology, University of Glasgow, UK) [29] to generate a gene correlation heat map. In addition, the co-expression networks of *PeAQP*s and sugar transport genes were constructed using Cytoscape 3.5.1 (Molecular Behavioral Neuroscience Institute, University of Michigan, Ann Arbor, MA, USA), based on the PCCs of the *PeAQP*s and sugar transport genes in the 26 moso bamboo samples [30]. The threshold for new edges was set with a PCC magnitude >0.70, which was believed to indicate strongly co-expressed genes.

### 2.6. Total RNA Isolation and qRT-PCR

For the diurnal variation expression analysis of the *PeAQP*s, the internodes at the base parts of the bamboo shoots were collected from the shoots that were used for root pressure measurements and quickly frozen into liquid nitrogen for RNA isolation. Total RNA isolation and qRT-PCR were carried out using previously described methods [8]. Primers for the quantitative analysis of *PeAQP*s were designed, and they are listed in Table S3. To ensure the reliability of the data, two reference genes, *TIP41* (Tonoplast Intrinsic Protein gene, GenBank: FP092936.1) and *NTB* (Nucleotide Tract-Binding protein gene, GenBank: FP091427.1) were used to analyze the expression levels of the *PeAQP*s. Both *NTB* and *TIP41*, which were found to be homogeneously expressed and which were adequate for normalization purposes, were used for gene expression analysis in moso bamboo [8,19,31]. All of the qRT-PCR steps followed the rules of minimum information for publication of quantitative real-time PCR experiments (MIQE) [32].

### 2.7. In Situ Hybridization

To investigate aquaporin gene expression in moso bamboo, the two-year-old seedlings were used for in situ hybridization. Moso bamboo culm and root tissue preparation and in situ hybridization were conducted according to the method described in [33]. Two candidate aquaporin genes (*PeTIP4;1* and *PeTIP4;2*) showing positive and negative correlations with the root pressure, respectively, were selected for tissue localization of expression analysis in moso bamboo culm and root cells. The gene-specific probe of *PeTIP4;1* was 5′-AGCCGCCCCCGGCAACTCCGGCGACCCG-3′, and that of *PeTIP4;2* was 5′-GGCCCATGATCGTATCGGTGCCGCCG-3′, which were complementary to the corresponding messenger RNAs (mRNAs) of *PeTIP4;1* and *PeTIP4;2*. The specific oligonucleotide probes were labeled with fluorescein isothiocyanate (FITC). A blue-fluorescent DNA stain of 4′,6-diamidino-2-phenylindole (DAPI) was used to stain double-stranded DNA (dsDNA) as a control. The images were captured with an IX71 fluorescence inverted microscope (Olympus, Tokyo, Japan). 

## 3. Results

### 3.1. Identification and Classification of Bamboo Aquaporin Genes

**Figure 1:**
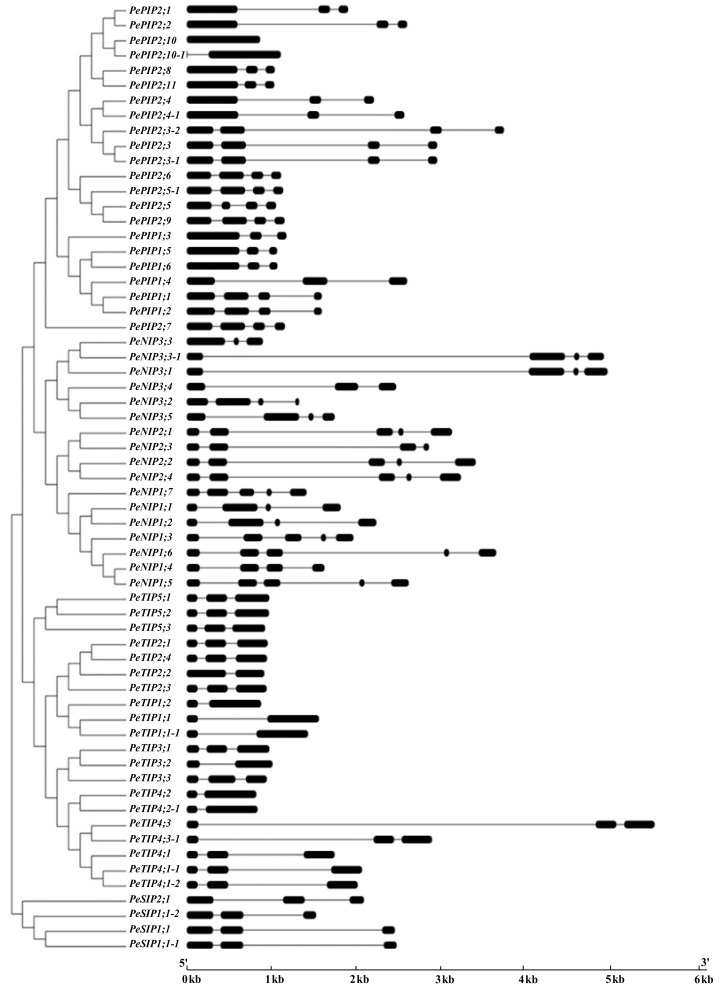
Phylogenetic relationships and gene structures of aquaporin (*AQP*) genes in moso bamboo. Round-corner rectangles and lines represent exons and introns, respectively. The x-axis represents the length of genes.

With the help of the updated genome and the transcriptomic data of moso bamboo [20], the authors performed a comprehensive analysis and identified aquaporin genes in 63 loci, of which 26 genes were identified by a previous study [8]. The newly-identified candidate genes were named, followed by the known *AQP* genes in bamboo [8]. Of these, some sequences only showed a small number of amino acid differences, deemed as single nucleotide polymorphism (SNP), and they were labeled as -1 or -2 at the end of the gene name. For example, PH02V02Gene21665.t1 and PH02V02Gene40544.t1 in the GABR showed two and five amino acid differences from PH01003141G0240 (PePIP2;3) in BambooGDB (International Center for Bamboo and Rattan, Beijing, China), and they were named as PePIP2;3-1 and PePIP2;3-2, respectively (Table S4). As a result, 63 aquaporin genes were obtained, including 22 *PePIP*s, 20 *PeTIP*s, 17 *PeNIP*s, and 4 *PeSIP*s. Detailed information, including BambooGDB (http://www.bamboogdb.org/, 5 March 2014) [34] and GABR database (http://gigadb.org/dataset/100498, 1 October 2018) [20] locus IDs, complementary DNA (cDNA) clone accession numbers, protein characteristics, phylogenetic analysis, subcellular localization, and transmembrane domains prediction, are listed in Table S4. The gene structures of each *PeAQP*s is shown in Figure 1, and these were similar to a previous study [8], in addition to the increased number of gene members.

Not only did the total members of the *PeAQP*s become bigger after the dramatically improved bamboo genome assembly, but the number of subgroups was also increased. For example, five subgroups of *PeTIP*s are found in the updated bamboo genome database, of which *PeTIP3* and *PeTIP5* subgroups were absent in the previous study [8]. However, members in the subfamily of XIPs were still not found in the updated genome, which further confirmed that XIPs do not exist in moso bamboo and other monocots. 

### 3.2. Phylogenetic Analysis of AQPs

**Figure 2:**
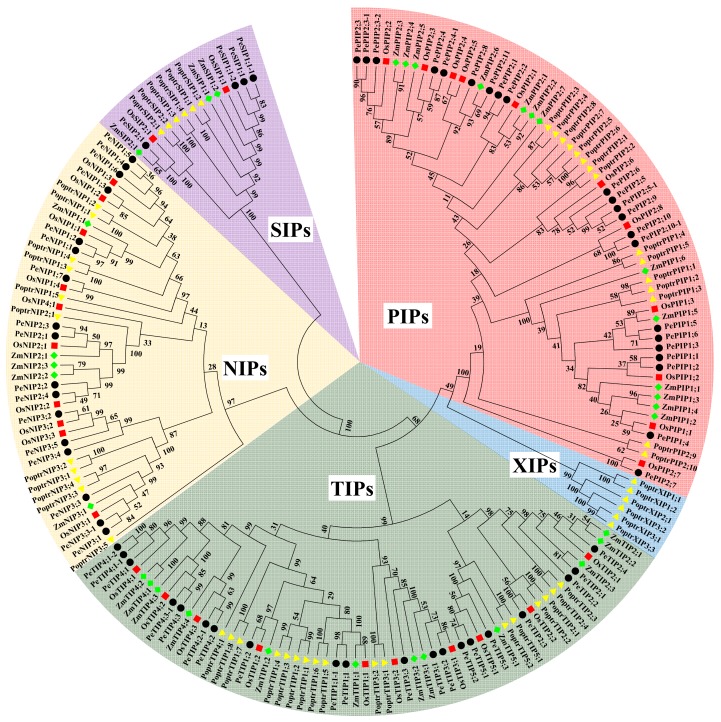
Phylogenetic analysis of the AQPs in *Phyllostachys edulis*, *Zea mays*, *Oryza sativa*, and *Populus trichocarpa*. Predicted amino acid sequences were aligned using the ClustalW2 program (Genome Campus, Cambridgeshire, UK), and the phylogenetic tree was constructed using the bootstrap neighbor-joining tree method (1000 replicates) in MEGA6.0 software. PIPs, SIPs, NIPs, TIPs and XIPs represented the different subfamilies of AQPs respectively. No sequences of bamboo were clustered into the clade of XIPs. The AQPs from *Phyllostachys edulis*, *Zea mays*, *Oryza sativa*, and *Populus trichocarpa* are indicated with prefixes of Pe, Zm, Os, and Pt, respectively.

To investigate the phylogenetic relationships of PeAQPs, a phylogenetic tree based on the AQP sequences of four plants, including three monocots (*P. edulis*, *Z. mays*, and *O. sativa*) and one dicot (*Populus trichocarpa*), was constructed. The results showed that they were clustered into five clades representing five distinct AQPs subfamilies. The XIPs in *P. trichocarpa* were clustered into a single clade. This further indicated that XIPs do not exist in moso bamboo or in other monocots. Consequently, the PeAQPs were distributed in four subfamilies, including 22 PePIPs, 20 PeTIPs, 17 PeNIPs, and 4 PeSIPs (Figure 2). In addition, the AQP members of monocots were clustered in closer branches, and those of moso bamboo had a close relationship with those of rice, respectively, which were consistent with previous research [8].

### 3.3. *PeAQP* Expression Pattern Analysis in Bamboo Tissues

**Figure 3:**
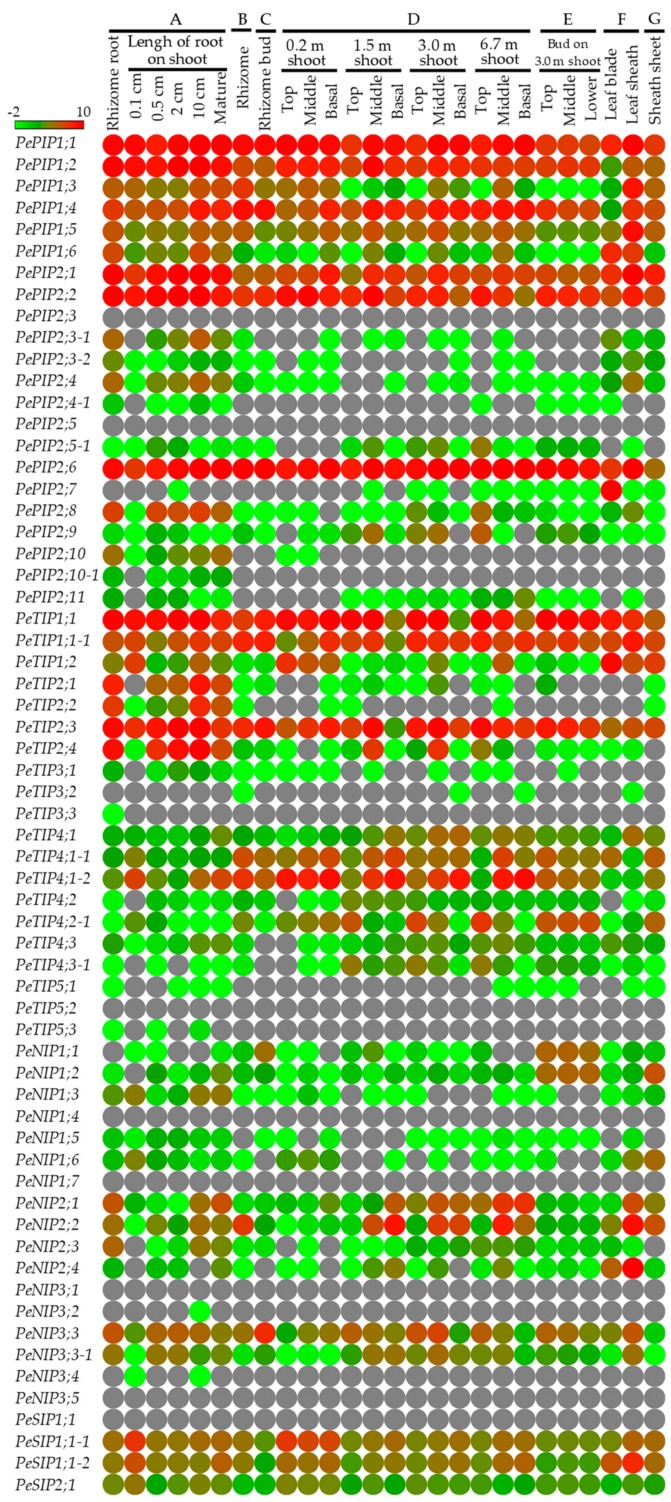
Expression profiles of *PeAQP*s in different tissues of moso bamboo. (**A**) Roots formed on rhizomes and those generated from the base of shoots of 0.1/0.5/2/10 cm in length, and mature roots with lateral roots; (**B**) Rhizomes; (**C**) Rhizome buds; (**D**) Shoot samples collected from the top/middle/basal portion of shoots at four development stages (0.2, 1.5, 3.0, and 6.7 m, respectively); (**E**) Buds, bud formed on the top/middle/basal portions of 3.0 m shoots; (**F**) Leaves including leaf blades and leaf sheaths; (**G**) Sheath sheets. The heat map was established using Matrix2png software. The scale represents the signal intensity of FPKM values after normalization by log_2_.

The recent transcriptome data generated from 26 different tissue samples collected from six main moso bamboo-producing areas in China were used to investigate the expression patterns of *PeAQP*s (Figure 3). Since most plants imbibe moisture through their roots, the authors investigated the changes of *PeAQP* expression at different stages of developing roots formed on bamboo shoots. Fifteen *PeAQP*s, including nine *PIP*s (*PePIP1;4*, *PePIP1;5*, *PePIP1;6*, *PePIP2;1*, *PePIP2;2*, *PePIP2;3-1*, *PePIP2;4*, *PePIP2;6*, and *PePIP2;10*), five *TIP*s (*PeTIP1;1*, *PeTIP2;1*, *PeTIP2;2*, *PeTIP2;3*, and *PeTIP2;4*) and one *NIP* (*PeNIP2;3*), showed an increasing trend with the continuous elongation of roots. Among these, the expression of *PePIP2;1*, *PePIP1;6*, and *PePIP2;6* in 10 cm roots was significantly up-regulated to 17.7, 13.7, and 12.9 times, respectively, compared to that in 0.1 cm roots. Additionally, no or weak transcript accumulation (fragments per kilobase of exon per million fragments mapped (FPKM) value <1) of *PePIP2;3-1*, *PePIP2;4*, *PePIP2;10*, *PeTIP2;1*, *PeTIP2;2*, *PeTIP2;4*, and *PeNIP2;3* was found in 0.1 cm roots, whereas they were all up-regulated significantly with root elongation. Accordingly, the results indicated that these genes might be involved in water absorption in roots.

Water is essential to sustain bamboo shoot growth in the boosting stage. In the top part of the shoots, five *PIP*s (*PePIP1;4*, *PePIP1;5*, *PePIP2;6*, *PePIP2;8*, and *PePIP2;9*), eight *TIP*s (*PeTIP1;1*, *PeTIP1;1-1*, *PeTIP2;3*, *PeTIP2;4*, *PeTIP4;1*, *PeTIP4;2-1*, *PeTIP4;3*, and *PeTIP4;3-1*), and one *NIP* (*PeNIP2;1*) had an increasing expression trend with the growth of bamboo shoots. In the middle part of the shoots, most of genes first showed a rise, followed by a decline, including six *PIP*s (*PePIP1;2*, *PePIP1;5*, *PePIP2;1*, *PePIP2;5-1*, *PePIP2;6*, and *PePIP2;9*), seven *TIP*s (*PeTIP1;1*, *PeTIP1;1-1*, *PeTIP2;3*, *PeTIP2;4*, *PeTIP4;1*, *PeTIP4;2*, and *PeTIP4;3-1*), and two *NIP*s (*PeNIP3;3* and *PeNIP3;3-1*). In the base part of different heights of shoots, six *PIP*s (*PePIP1;2*, *PePIP1;3*, *PePIP1;5*, *PePIP1;6*, *PePIP2;1*, and *PePIP2;2*), four *TIP*s (*PeTIP1;1*, *PeTIP1;2*, *PeTIP2;3*, and *PeTIP4;2-1*), and two *SIP*s (*PeSIP1;1-1* and *PeSIP2;1*) were highly expressed in 0.2 m shoots, whereas most of them demonstrated a decreasing expression trend with an increase of shoot height. Moreover, some genes were found to have the characteristics of tissue-specific expression. For example, *PeNIP2;4* showed a higher expression level in leaf sheaths; *PePIP2;7* was exclusively expressed in leaves. These tissue-specific *PeAQP*s might have specific functions in those tissues.

### 3.4. Correlation Analysis of the Root Pressure and PeAQP Expression in Bamboo Shoots

**Figure 4:**
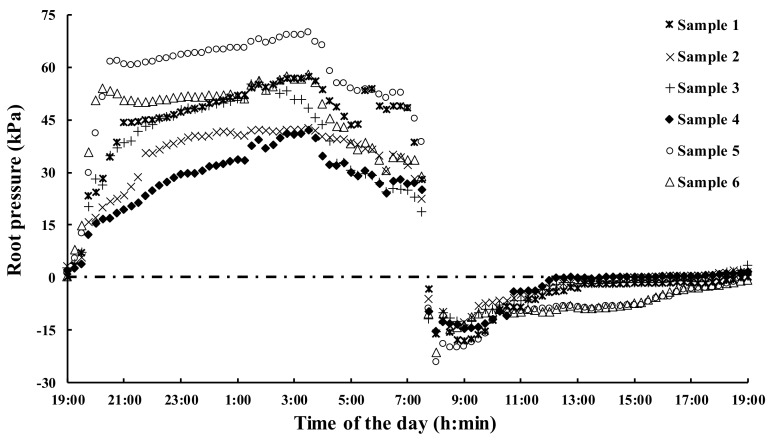
Diurnal variations in the root pressure of moso bamboo. The measurement was conducted from 7:00 p.m. on 20 April 2017 to 7:00 p.m. on 21 April 2017. The root pressure values from six individual moso bamboo shoots at ~6 m height were measured.

The measurement of root pressure was conducted; the pressure displayed diurnal rhythm changes, which were negative in the daytime and positive at night. The root pressure of bamboo shoots presented a continued upward trend during the period of 7:00 p.m. to 3:30 a.m. and remained positive until 7:30 a.m. The pressure decreased sharply to negative at 7:45 a.m., which was two hours after sunrise. Those values remained negative, which showed a decreased trend at first, and then they gradually recovered to zero and finally rose to positive values at 7:00 p.m. The maximum value of the root pressure was approximately 69.9 kPa at 3:00 a.m., which could pump water up to 7 m in height (Figure 4). As is known, truncated damage is deadly to bamboo shoots; only half of bamboo shoots can be used for further measurement in the second day. Although the values of root pressure became weaker in the second day, they still showed similar trends to the first day (Figure S2).

**Figure 5:**
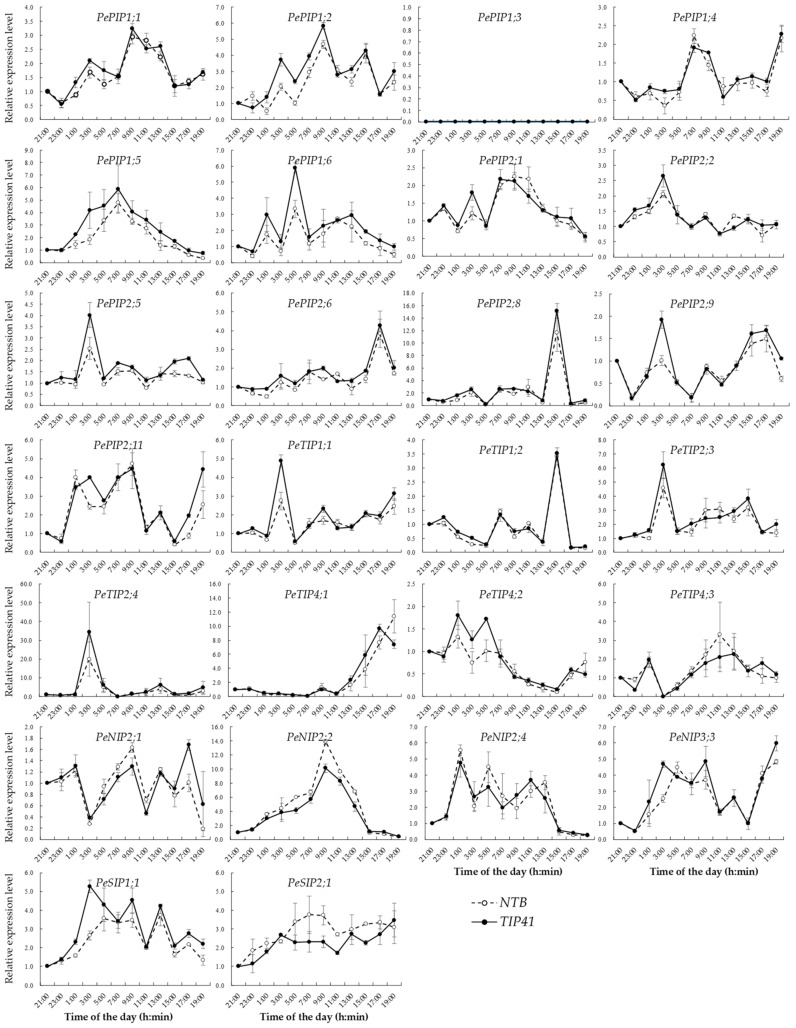
The daily changes of the relative expression levels of 26 *PeAQP*s were determined by qRT-PCR. The relative expression level of each gene in the sample at 21:00 was used as a calibrator. Gene expression was calculated with the 2^−ΔΔCt^ method, using both *NTB* and *TIP41* as the reference genes.

Meanwhile, 26 *PeAQP*s, with FPKM values in any part of the shoot that were greater than 10, were considered as taking part in the regulation of root pressure, and these were selected for further analyses. The combined analyses of the gene expression and root pressure changes in bamboo shoots with diurnal rhythm were conducted. The results indicated that all of the selected genes were detected in the bamboo base part of the shoots, except *PePIP1;3*, and most of them showed diurnal rhythm changes (Figure 5). For example, *PePIP2;8* and *PeTIP1;2* presented a peak value at 3:00 p.m., and the peak value of *PePIP2;6* was at 5:00 p.m. Furthermore, *PePIP1;4*, *PeTIP4;1*, and *PeNIP3;3* exhibited an expression peak at 7:00 p.m. These results indicated that they may take part in the positive control of root pressure. Meanwhile, *PePIP1;1*, *PePIP1;2*, and *PePIP1;4* demonstrated expression peaks at 7:00 a.m., those of *PePIP1;5* and *PeNIP2;2* at 9:00 a.m., and *PePIP2;1* was expressed at a higher level in the period from 7:00 a.m. to 9:00 a.m., indicating that they might be involved in the negative control of root pressure.

**Figure 6:**
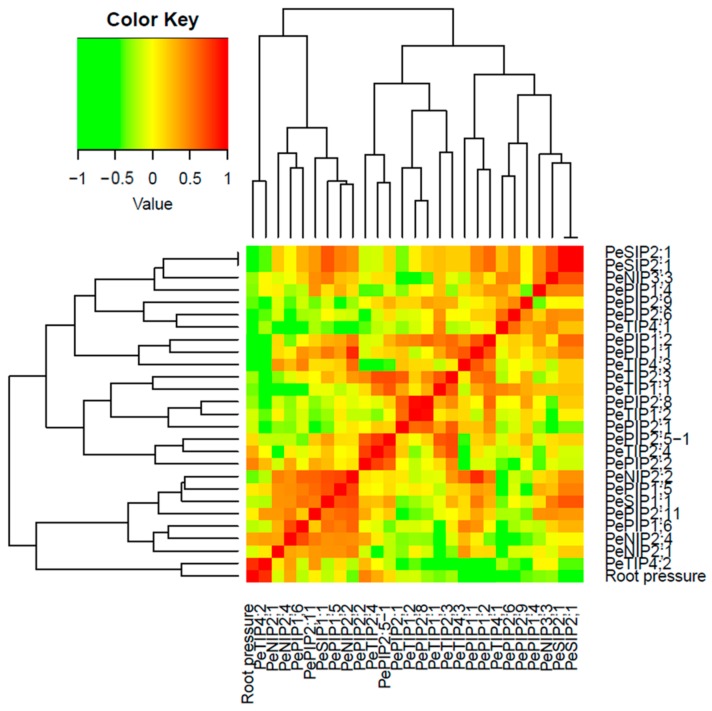
Correlation analyses of the expression levels of *PeAQP*s and the values of root pressure.

Based on the correlation analyses of aquaporin gene expression levels and the root pressure values, the authors found that the expression levels of *PeTIP4;2* and *PePIP2;2* showed significant positive correlation with the root pressure, and the values of Pearson’s correlation were 0.838 and 0.603, respectively. Conversely, the expression levels of *PeTIP4;3*, *PeTIP4;1*, *PePIP1;1*, and *PePIP1;2* presented significant negative correlations with the root pressure, and the values were 0.689, 0.508, 0.560, and 0.504, respectively (Figure 6). These results indicated that all of the above genes might participate in the regulation of root pressure through gene expression.

### 3.5. Co-Expression Network Analyses of PeAQPs and Sugar Transport Genes

During the bamboo fast-growth stage, non-structural carbohydrates (NSCs), such as sugars and starch, provided a temporary source of carbon for the bamboo shoots, since their photosynthesis could not meet the immediate carbon demands. Almost all of the NSCs were transferred to new “explosively growing” bamboo shoots from the mature bamboos via underground rhizomes, for the structural growth and metabolism of shoots [3]. Therefore, the expression of sugar transport genes was investigated, and the co-expression relationship of *PeAQP*s and sugar transport genes in bamboo shoots was further analyzed. 

The results indicated that a total of 86 sugar transport genes were identified in the updated genome of moso bamboo, including 7 putative monosaccharide sensing protein (AZT/MSSP) genes, 4 myo-inositol transporter (ITR/MIT) genes, 5 hexose transporter (STP/HXT) genes, 5 plastidic glucose transporter (pGlcT) genes, 19 polyol transporter (PLT) genes, 2 sugar facilitator (SFP) genes, 8 sucrose transporter (SUC/SUT) genes, and 36 sugars which will eventually be exported transporter (SWEET) genes (Table S2). Conversely, members of the vacuolar glucose transporter (VGT) gene family were still not found in this updated genome. In addition, the expression levels of these sugar transport genes were also analyzed, and the heat map demonstrated a diversity of expression patterns (Figure S1).

**Figure 7:**
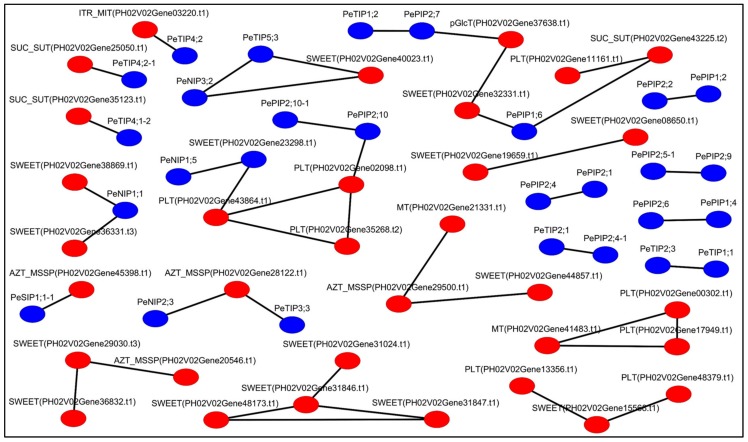
Gene co-expression network analyses of *PeAQP*s and sugar transport genes. Red nodes and blue nodes represent *PeAQP*s and sugar transport genes, respectively.

The authors next investigated the relationships of *PeAQP*s and sugar transport genes for bamboo shoot development. The 86 sugar transport genes and 63 *PeAQP*s genes were used to build a co-expression network for inferring their function in bamboo shoot development. A total of 33 sugar transport genes and 27 *PeAQP*s were found to have positive features of co-expression (Figure 7). In the network, *PePIP2;10*, *PePIP2;10-1*, and *PeNIP1;5* were co-expressed with *PLA* and *SWEET* genes. *PeTIP4;1-2* and *PeTIP4;2-1* were co-expressed with *SUC*/*SUT* genes. *PePIP1;6*, *PePIP2;7*, and *PeTIP1;3* were co-expressed with four types of sugar transporter genes, including *PLT*, *SUC*/*SUT*, *SWEET*, and *pGlcT*. These results were similar to those co-expressed genes involved in water and sugar transport-related processes in the developing grass stems of *Setaria viridis* [35], which indicated that the *PeAQP*s might participate in the regulation of bamboo shoot growth through water and sugar transport, which would require further studies.

### 3.6. In Situ Hybridization of PeAQPs

**Figure 8:**
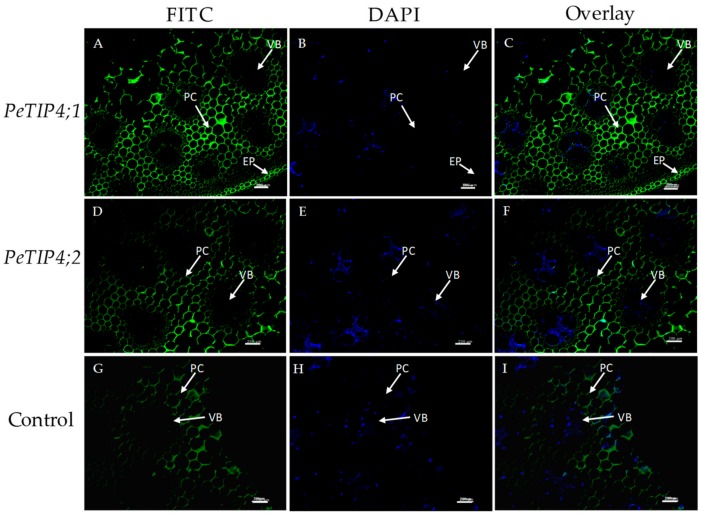
Fluorescence in situ hybridization of *PeTIP4;1* and *PeTIP4;2* in the culms of moso bamboo. (**A**,**D**,**G**): images with fluorescein isothiocyanate (FITC) only; (**B**,**E**,**H**): images with 4′,6-diamidino-2-phenylindole (DAPI) only; (**C**,**F**,**I**): merged images. EP: epidermis, VB: vascular bundle, PC: parenchyma cell. Bar  =  200 μm.

**Figure 9:**
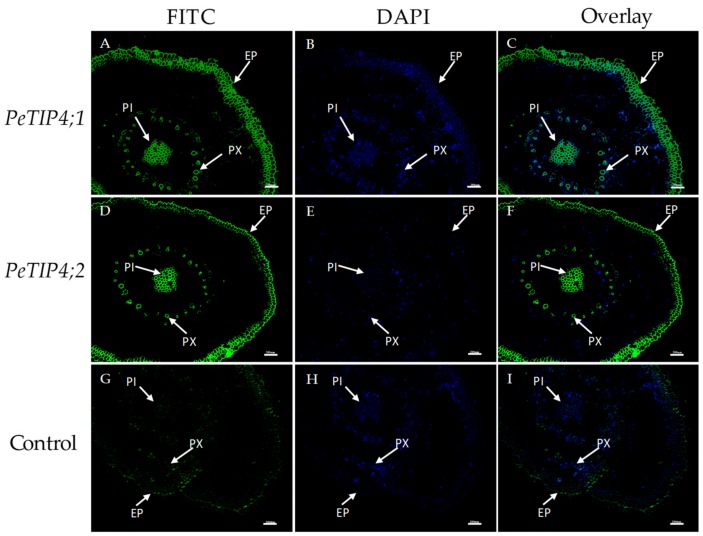
Fluorescence in situ hybridization of *PeTIP4;1* and *PeTIP4;2* in the roots of moso bamboo. (**A**,**D**,**G**): images with FITC only; (**B**,**E**,**H**): images with DAPI only; (**C**,**F**,**I**): merged images. EP: epidermis, PI: pith, PX: primary xylem. Bar  =  200 μm.

Two candidate aquaporin genes, *PeTIP4;1* and *PeTIP4;2*, and belonging to the same subfamily, were selected for the tissue localization of expression analysis using in situ hybridization. The results showed that both *PeTIP4;1* and *PeTIP4;2* were mainly expressed in the parenchyma and epidermal cells, which were the most active cells in the bamboo culm of young seedlings (Figure 8). This is consistent with the fact that the parenchyma cells surrounding the vascular bundle are the main water storage sites of the bamboo culm [36]. Although the vascular bundle is the main point of water transport, the expression level in the vascular bundles was relatively lower. Furthermore, to better illustrate the expression of the two genes, the authors performed in situ hybridization of *PeTIP4;1* and *PeTIP4;2* in root tissues. The results demonstrated that both *PeTIP4;1* and *PeTIP4;2* were mainly expressed in the epidermis, pith, and primary xylem of the roots (Figure 9). The epidermis and primary xylem were the main points of absorption and transport in roots, respectively [37]. These results indicated that *PeTIP4;1* and *PeTIP4;2* might play important roles in water absorption and transport, and they may also take part in the regulation of root pressure.

## 4. Discussion

Bamboos are a category of fast-growing plants, which can grow with surprising speed at their growth spurt stage in spring. During the fast-growth period, bamboos need a large quantity of water and energy to supply cell division and elongation, indicating that a strong water potential gradient is needed to maintain the diurnal water balance, and to drive fast growth. AQPs have the function of regulating the rapid flow of water in cells, and approximately 70–90% of the water flow in cells is completed by AQPs [38]. The remobilized soluble sugars are the necessary carbon source to satisfy the carbon demand of the fast-growing bamboo shoots, and they must be transferred from the mother bamboo, since the new shoots have almost no photosynthesis capability [39]. Therefore, water supply and sugar transport are very important for bamboo shoot growth. With the rapid development of sequencing technologies that are used for the cognition of the plant genome, an increasing number of plant genomes have been re-sequenced. More and more members of the aquaporin family with higher sequence similarities have been found in different plant species, which facilitated the authors’ aim to determine the members of the aquaporin family and the sugar transport gene families for further study of their functions in plants (Table S1).

In this study, the authors expanded the members of *PeAQP*s from the 26 to 63, based on the updated genome of moso bamboo generated by the GABR Project, using both Illumina and PacBio sequencing platforms, of which the member number exceeded the numbers in most monocots and dicots. The number was closer to those of two monocots, *Musa acuminate* (57) and *Panicum virgatum* (68), and one dicot, *Populus trichocarpa* (58). It has been validated that the genome of moso bamboo is 2.05 Gbp, and that it consists of 24 pairs of chromosomes (2*n* = 48) and that it is characteristic of a diploid. It was the whole-genome duplication event of moso bamboo during evolution that led to the generation of more gene duplicates in gene families [2]. Consistent with the previous study, all of the 63 *PeAQP*s were still classified into four subfamilies. Although the number of gene members was significantly increased, members of the XIP subfamily were still not found in the updated moso bamboo genome, which further confirmed that XIPs are not present in moso bamboo and other monocots. Additionally, members of TIP3 and TIP5 were found in the genome, which were absent in a previous study [8]. After the expression analyses, the authors found that all of the newly discovered *PeAQP*s showed lower-expressed levels, indicating that a number of lower expression genes were found in the dramatically improved genome assembly.

By comparing the gene structures of the *PeAQP*s, the authors found that some genes have very long intron regions, such as in *PeTIP4;3* and *PeNIP3:3-1*, whereas the corresponding genes in the same subfamily, such as *PeTIP4;3-1* and *PeNIP3:3*, have small intron regions. From the transcription expression profile, they found that *PeTIP4;3* has a higher expression level in roots, and a lower expression level in the top part of the shoots, compared to that of *PeTIP4;3-1*. *PeNIP3:3* has a higher expression level in almost all tissues, compared to that of *PeNIP3:3-1*. The two homologous genes with different intron lengths in the same subfamily have different expression patterns, which might be explained by the introns regulating the gene transcripts in different ways. Although the intron regions are spliced out during transcription, introns have increasingly been shown to serve important biological functions. It has been confirmed that introns can improve the expression of exogenous genes in transgenic plants [40,41]. The status of *PeAQP*s in bamboo still needs further study.

Most of the water supply in bamboo needs to be absorbed by roots from the soil, and then transported upwards, for the growth of bamboo shoots. The expression levels of *PeAQP*s in different tissues, as well as those at different developmental stages, may be a cue to reflect their physiological functions. Almost all of the *PeAQP*s were detected in all samples, which indicated that they might be essential genes in different tissues, and in different development stages. For example, with the elongation of bamboo roots, nine *PIP*s (*PePIP1;4*, *PePIP1;5*, *PePIP1;6*, *PePIP2;1*, *PePIP2;2*, *PePIP2;3-1*, *PePIP2;4*, *PePIP2;6*, and *PePIP2;10*), five *TIP*s (*PeTIP1;1*, *PeTIP2;1*, *PeTIP2;2*, *PeTIP2;3*, and *PeTIP2;4*), and one *NIP* (*PeNIP2;3*) showed increasing expression trends, which indicated they might have main functions in the transportation and absorption of water in the process of root maturation. In addition, with the development of bamboo shoots, the expression of five *PIP*s (*PePIP1;4*, *PePIP1;5*, *PePIP2;6*, *PePIP2;8*, and *PePIP2;9*), eight *TIP*s *(PeTIP1;1*, *PeTIP1;1-1*, *PeTIP2;3*, *PeTIP2;4*, *PeTIP4;1*, *PeTIP4;2-1*, *PeTIP4;3*, and *PeTIP4;3-1*), and one *NIP* (*PeNIP2;1*) was increased in different parts of shoots. All of those genes might be involved in water transportation and absorption in bamboo shoots. 

The previous study of bamboo showed that a decline in nocturnal xylem pressure, as measured from the base to the apex of individual culms, was very close to the gradient that was expected by hydrostatic pressure, which confirmed that the origin of the nocturnal xylem pressure was at the root [9]. In addition, there was little transpiration pull in the bamboo shoots, and the driving force for water transport in the daytime mainly originated from the root pressure. Therefore, water transport in the bamboo shoots was related closely to root pressure. The measurement of root pressure showed a periodical change of water transport from the rhizome network to the shoots overnight, to sustain water loss in the daytime. In general, the value of the root pressure was larger at night, which became weak and then turned negative in the daytime. The diurnal variations of root pressure measured in another growing season was consistent with this fact (Figure S3).The growth rate overnight was faster than that in the daytime. The qRT-PCR results showed that the expression levels of some *PeAQP*s exhibited significant positive correlations with the root pressure, and some *PeAQP*s presented significant negative correlations with root pressure, indicating that these genes might play crucial roles in the regulation of water supply for cell division and elongation during the fast growth of bamboo shoots.

It has been reported that the maximum size in bamboo species is determined below ground by the maximum pressure that can be generated in roots overnight, and that water transport in bamboo is dependent on root pressure to repair the hydraulic dysfunction that is sustained during normal diurnal gas exchange [9]. Sugars are the main photosynthesis products, and they can widely take part in the synthesis of lignin, cellulose, hemicelluloses, and pectin, as well as play essential roles in growth, germination, and resistance to stresses [3]. Soluble sugars were often dissolved in water to reduce the water potential of plant cells, which resulted in water flowing backward along the water potential gradient. Hence, the expression of the sugar transport genes and *PeAQP*s was very important for water transport during the bamboo fast-growth stage.

New bamboo shoots have little photosynthetic capability, and most of the required carbon source for fast growth must be transferred to the shoots by the sugar transporter from the mother plant [39]. Sucrose is one of the important carbon sources, and both the tonoplast-localized and the plasma membrane-localized SUTs can contribute to the transport of sucrose which is used as osmotic substances to control the hydrostatic pressure [42,43]. The plasma membrane-localized SWEETs and SUTs may have a role in transporting sugar into the apoplast [44]. Many members of *PeAQP*s were co-expressed positively with sugar transport genes, such as *PeTIP4;1-2* and *PeTIP4;2-1* co-expressed with SUC/SUT genes, *PeNIP1;1* co-expressed with SWEET genes, and *PePIP1;6* and *PePIP2;7* co-expressed with both SUC/SUT and SWEET genes in moso bamboo shoots, indicating that aquaporins and sugar transporters might work together for the regulation of water and carbon source supply during the period of shoot rapid growth, through gene co-expression.

Although woody bamboo had excellent mechanical strength, the bamboo lacked secondary growth and had a hollowed culm. Bamboo root pressure was used to recharge the culm water storage, specifically the culm parenchyma cells surrounding all vascular bundles [45]. Two root pressure-correlated genes, *PeTIP4;1* and *PeTIP4;2*, were selected to investigate their expression in the culms of moso bamboo seedlings by in situ hybridization. The results showed that they were all mainly expressed in the parenchyma cells of the culm, which agrees with evidence that parenchyma cells serve as a large storage site for water (∼52% of the bamboo culm) [36]. The expression signal was also stronger in the epidermal cells of young culms. Additionally, the authors also found that *PeTIP4;1* and *PeTIP4;2* were highly expressed in the epidermis, pith, and primary xylem of the root. *PeTIP4;1-1* could increase the length of taproots in transgenic *A. thaliana* under osmotic stresses, and it could enhance the tolerance of transgenic plants to osmotic stresses [19]. This indicates that *PeTIP4;1-1* has the ability to regulate the water osmotic balance in roots. Taking this into consideration, the authors find that *PeTIP4;1* and *PeTIP4;2* might not only participate in the formation of root pressure, but they may also play important roles in water absorption and transport in the root and culms. Therefore, they believe that *PeTIP4;1* and *PeTIP4;2* must be the key genes for the formation of root pressure, water absorption, and transportation in bamboo. However, the regulation of bamboo fast growth is a complex network, and many studies need to be carried out for a further understanding of the functions of *PeAQP*s.

## 5. Conclusions

Water and energy supply are essential for cell division and elongation, resulting in the rapid growth of woody bamboo, which will affect the yield and quality of bamboo. AQPs have the function of regulating the rapid flow of water in cells, whereas sugar transport genes are responsible for the transport of soluble sugars, providing both energy and a low water potential for cells during the explosive growth of new bamboo shoots. The expression levels of many *PeAQP*s exhibited significant correlations with the root pressure, and many sugar transport genes were positively co-expressed with multiple types of *PeAQP*s. These findings indicate that aquaporins and sugar transporters might work together for the regulation of water supply through gene co-expression in moso bamboo, which increases the understanding of the aquaporin-led mechanism for bamboo fast growth.

## Supplementary Materials

The following are available online at http://www.mdpi.com/2073-4409/7/11/195/s1. Table S1: Aquaporin genes in 25 plant species, Table S2: Information on sugar transport genes in moso bamboo, Table S3: Primers used for qRT-PCR, Table S4: Nomenclature and protein information on AQPs in moso bamboo, Figure S1: Expression profiles of sugar transport genes in different tissues of moso bamboo. (A) Roots formed on rhizomes, and those generated from the base of shoots with 0.1/0.5/2/10 cm length and mature roots with lateral roots; (B) Rhizomes; (C) Rhizome buds; (D) Shoot samples collected from the top/middle/basal portions of shoots at four development stages (0.2, 1.5, 3.0, and 6.7 m, respectively); (E) Buds, buds formed on the top/ middle/basal portions of 3.0 m shoots; (F) Leaves, including leaf blades and leaf sheaths; (G) Sheath sheets. The heat map was established using Matrix2png software. The scale represents the signal intensity of FPKM values after normalized by log_2_, Figure S2: Diurnal variations in the root pressure of moso bamboo shoots in the second day (7:00 p.m. on 21 April 2017 to 7:00 p.m. on 22 April 2017). The truncated bamboo shoots were measured, and only half of the bamboo shoots could be used in the second day, which the values of root pressure were much lower than those measured in the first day, Figure S3: Diurnal variations in the root pressure of moso bamboo shoots measured in another growing season (Pre-experiment).
