# Supplementary material for: Whole-Genome and Expression Analyses of Bamboo Aquaporin Genes Reveal Their Functions Involved in Maintaining Diurnal Water Balance in Bamboo Shoots
Source: Cells. 2018 Nov 2;7(11):195. doi: 10.3390/cells7110195 (PMC6262470; doi:10.3390/cells7110195)
Supplement: Supplementary file 1 [file cells-07-00195-s001.zip › supplementary files/Supplementary Materials Table S1, S3, S4, and Figure S1, S2, S3.docx]

Table S1: Aquaporin genes in 25 plant species.

| Plant species | PIPs subfamily | TIPs  subfamily | NIPs  subfamily | SIPs  subfamily | XIPs  subfamily | Total | Reference |
| --- | --- | --- | --- | --- | --- | --- | --- |
| *Arabidopsis thaliana* | 13 | 10 | 9 | 3 | 0 | 35 | Johanson et al., 2001 |
| *Beta vulgaris* | 7 | 8 | 9 | 3 | 1 | 28 | Kong et al., 2017 |
| *Brachypodium distachyon* | 10 | 10 | 7 | 1 | 0 | 28 | Azad et al., 2016 |
| *Brassica napus* | 43 | 35 | 32 | 11 | 0 | 121 | Yuan et al., 2017 |
| *Brassica rapa* | 20 | 14 | 13 | 6 | 0 | 53 | Tao et al., 2014 |
| *Cicer arietinum* | 8 | 13 | 15 | 4 | 0 | 20 | Deokar and Tar'an, 2016 |
| *Hevea brasiliensis* | 15 | 17 | 9 | 4 | 6 | 51 | Zou et al., 2015 |
| *Hordeum vulgare* | 11 | 7 | 4 | 2 | 0 | 22 | Hove et al., 2015 |
| *Jatropha curcas* | 9 | 9 | 8 | 4 | 2 | 32 | Zou et al., 2016 |
| *Linum usitatissimum* | 16 | 17 | 13 | 2 | 3 | 51 | Shivaraj et al., 2017 |
| *Manihot esculenta* | 14 | 13 | 10 | 4 | 4 | 45 | Putpeerawit et al., 2017 |
| *Musa acuminate* | 18 | 17 | 9 | 3 | 0 | 57 | Hu et al., 2015b |
| *Oryza sativa* | 11 | 10 | 10 | 2 | 0 | 33 | Sakurai et al., 2005 |
| *Panicum virgatum* | 21 | 20 | 23 | 4 | 0 | 68 | Azad et al., 2016 |
| *Phaseolus vulgaris* | 12 | 13 | 10 | 4 | 2 | 41 | Ariani and Gepts, 2015 |
| *Phyllostachys edulis* | 10 | 6 | 8 | 2 | 0 | 26 | Sun et al., 2016a |
| *Physcomitrella patens* | 9 | 4 | 5 | 2 | 2 | 23 | Danielson and Johanson, 2008 |
| *Populus trichocarpa* | 15 | 18 | 11 | 7 | 7 | 58 | Gupta and Sankararamakrishnan, 2009 |
| *Selaginella moellendorffii* | 3 | 3 | 8 | 1 | 3 | 19 | Anderberg et al., 2014 |
| *Setaria italica* | 12 | 15 | 12 | 3 | 0 | 42 | Azad et al., 2016 |
| *Setaria viridis* | 12 | 14 | 12 | 3 | 0 | 41 | McGaughey et al., 2016 |
| *Solanum tuberosum* | 15 | 11 | 10 | 3 | 8 | 47 | Venkatesh et al., 2013 |
| *Sorghum bicolor* | 13 | 11 | 11 | 3 | 0 | 38 | Azad et al., 2016 |
| *Solanum lycopersicum* | 14 | 11 | 12 | 4 | 6 | 47 | Reuscher et al., 2013 |
| *Zea mays* | 13 | 11 | 4 | 3 | 0 | 31 | Chaumont et al., 2001 |

Table S3: Primers used for qRT-PCR.

| Gene name | Forward Primer (5'---3') | Reverse Primer (5'---3') | Product size (bp) |
| --- | --- | --- | --- |
| *PePIP1;4* | AGTTGCCAGTGGCTACACCAAG | GGATAGGAACATGGGAGTCCCT | 126 |
| *PePIP1;5* | GCAGGAAGCTGTCCCTGACC | ATGTAGAGCGGCTGCTGGAAC | 106 |
| *PePIP1;6* | GCGGAAGCTGTCCCTCGT | TGCTGGAAACCCTTGACGAC | 93 |
| *PePIP2;8* | CTCTGCTCTACATGGTCGCG | CGTAGAACCCGCTCTGGAAC | 81 |
| *PePIP2;9* | GAAGGCATGGGACGACCACT | CGCCTTGATGGCTGCCTT | 106 |
| *PePIP2;10* | CGGCAATACTACTGCTAGCGGT | TGACGTAGACGAAGAGGAGCGT | 140 |
| *PePIP2;11* | CTGCTCTACATGGCAGCCCA | GCGTAGAACCCGCTCTGGA | 80 |
| *PeTIP2;3* | CGCTTACACTAAGTTGACGGGTG | TGGCGACCGCGACGAAC | 98 |
| *PeTIP2;4* | CGCTGGCAACTGGGTCTACT | TTATAAGGGCTCGTACTCCTGGT | 124 |
| *PeTIP3;1* | CCATCGTCGCCACCCTCCT | ATCACCGCCTCCAGCAGCA | 112 |
| *PeTIP3;2* | TACCTTGTCGCTAATCCTGCCAT | TCATCACCGCCTCCAGCAG | 133 |
| *PeTIP3;3* | CGCGTCATTATATGCGTGATGT | GACCCAGTACACCCGATGGTT | 209 |
| *PeTIP4;3* | GGTGCCTCACCGGTGGAAT | CGTGAAGGTGAACACGGCCT | 98 |
| *PeTIP5;1* | TTCGGGCTCTTCACCGCGT | GAGATACCCAGTAGAAGATGGCGCT | 130 |
| *PeTIP5;2* | GCTGCCGTGTTCATCGCC | CCGAGCAGTTGAGACATCCAG | 128 |
| *PeTIP5;3* | CCTCTACTGGGCCTCCCAGT | TCATGTCCACGGCAACCCT | 107 |
| *PeNIP1;4* | AGGCCGTAGGAGGTGTGGTGT | TTACGACGACGCTCGGATCC | 104 |
| *PeNIP1;5* | GCAGCACGAGCAATCACCAC | TACCGGCCGTCCTCAGGTT | 132 |
| *PeNIP1;6* | GGAATCAGCACCAGCAATCATC | AGCCGGCGAAGATGAGTAAGT | 97 |
| *PeNIP1;7* | AGGAGGTGGTGTATGATCACCAGT | TTCACTGTGATGGCTCCCAAG | 142 |
| *PeNIP2;1* | CTACAACGACAGATTGCTCGTG | ACGAACACCAGCAAGAACGT | 150 |
| *PeNIP2;3* | AACGAGAGGTCGCTCGCA | ACGAACACCAGCAAGAACGT | 90 |
| *PeNIP2;4* | GGCGCGTGGGTCTACACTTACAT | TCGGCGGCGATATTGCACT | 119 |
| *PeNIP3;3* | CCTCGTGGCCGGGCCGAC | CAGCGTCGGCGCGATCAGGT | 115 |
| *PeNIP3;4* | GTGCTGTCCACCATCGTCAT | ACCCGGAGATGTGCACGAT | 121 |
| *PeNIP3;5* | TCTCTCATTCACCTCAACCTAGCT | CGCCGACAGAAGGATGAAGAT | 113 |

Table S4: Nomenclature and protein information on AQPs in moso bamboo.

| Name | Accession number in BambooGDB | Accession number in GABR | Accession number in NCBI | Protein length (aa) | Molecular weight (kD) | TM helices | pI | Plant-mPLoca |
| --- | --- | --- | --- | --- | --- | --- | --- | --- |
| PePIP1;1 | PH01000000G6020 | PH02V02Gene00515.t1 | FP099961.1 | 289 | 30.85 | 6 | 8.83 | plas |
| PePIP1;2 | PH01000272G0290 | PH02V02Gene26777.t1 |  | 289 | 30.91 | 6 | 9 | plas |
| PePIP1;3 | PH01000788G0340 | PH02V02Gene23370.t1 | FP097691.1 | 296 | 31.41 | 6 | 9.06 | plas |
| PePIP1;4 | PH01000272G0290 | PH02V02Gene43834.t1 |  | 283 | 30.16 | 6 | 8.49 | plas |
| PePIP1;5 |  | PH02V02Gene10933.t1 |  | 289 | 30.81 | 6 | 8.31 | plas |
| PePIP1;6 |  | PH02V02Gene16332.t1 |  | 289 | 30.77 | 6 | 8.31 | plas |
| PePIP2;1 | PH01001060G0550 | PH02V02Gene33713.t1 |  | 287 | 30.11 | 6 | 7.67 | plas |
| PePIP2;2 | PH01002412G0140 | PH02V02Gene19767.t1 | FP099351.1 | 287 | 30.08 | 6 | 6.95 | plas |
| PePIP2;3 | PH01003141G0240 |  | FP096147.1 | 288 | 30.46 | 6 | 7.64 | plas |
| PePIP2;3-1 |  | PH02V02Gene21665.t1 |  | 288 | 30.48 | 6 | 7.64 | plas |
| PePIP2;3-2 |  | PH02V02Gene40544.t1 |  | 288 | 30.42 | 6 | 8.57 | plas |
| PePIP2;4 | PH01000001G4670 | PH02V02Gene08461.t1 | FP097850.1 | 290 | 30.55 | 6 | 7.68 | plas |
| PePIP2;4-1 |  | PH02V02Gene23172.t1 |  | 292 | 30.77 | 6 | 7.69 | plas |
| PePIP2;5 | PH01000929G0130 |  |  | 220 | 23.55 | 4 | 8.81 | plas |
| PePIP2;5-1 |  | PH02V02Gene19140.t1 |  | 284 | 30.14 | 6 | 9.26 | plas |
| PePIP2;6 | PH01000450G0080 | PH02V02Gene26444.t2 | FP095552.1 | 282 | 29.94 | 6 | 9.22 | plas |
| PePIP2;7 | PH01000332G0830 | PH02V02Gene05107.t1 |  | 287 | 30.17 | 6 | 9.23 | plas |
| PePIP2;8 |  | PH02V02Gene19765.t1 |  | 286 | 29.94 | 6 | 8.65 | plas |
| PePIP2;9 |  | PH02V02Gene19144.t1 |  | 283 | 30.09 | 6 | 9.51 | plas |
| PePIP2;10 |  | PH02V02Gene44017.t1 |  | 293 | 30.74 | 6 | 9.33 | plas |
| PePIP2;10-1 |  | PH02V02Gene48710.t1 |  | 292 | 30.74 | 6 | 9.23 | plas |
| PePIP2;11 |  | PH02V02Gene33714.t1 |  | 288 | 29.98 | 6 | 8.31 | plas |
| PeTIP1;1 | PH01000157G0460 | PH02V02Gene03635.t1 | FP094394.1 | 250 | 25.81 | 6 | 6.01 | vacu |
| PeTIP1;1-1 |  | PH02V02Gene11054.t1 |  | 250 | 25.80 | 6 | 6.01 | vacu |
| PeTIP1;2 | PH01001117G0550 | PH02V02Gene34546.t1 | FP098066.1 | 252 | 25.41 | 6 | 5.61 | vacu |
| PeTIP2;1 | PH01000023G0610 | PH02V02Gene01978.t1 |  | 248 | 25.11 | 6 | 5.87 | vacu |
| PeTIP2;2 | PH01001207G0140 | PH02V02Gene44335.t1 |  | 272 | 27.31 | 6 | 6.39 | vacu |
| PeTIP2;3 |  | PH02V02Gene50341.t1 |  | 248 | 25.06 | 6 | 5.67 | vacu |
| PeTIP2;4 |  | PH02V02Gene42792.t1 |  | 250 | 25.22 | 6 | 5.54 | vacu |
| PeTIP3;1 |  | PH02V02Gene14868.t1 |  | 261 | 27.34 | 6 | 8.1 | vacu |
| PeTIP3;2 |  | PH02V02Gene36459.t1 |  | 201 | 21.48 | 4 | 6.64 | vacu |
| PeTIP3;3 |  | PH02V02Gene22760.t1 |  | 238 | 25.20 | 5 | 9.88 | vacu/plas |
| PeTIP4;1 | PH01000003G3730 | PH02V02Gene31034.t1 |  | 252 | 25.39 | 6 | 6.42 | vacu |
| PeTIP4;1-1 |  | PH02V02Gene26700.t1 | FP096242.1 | 251 | 25.48 | 6 | 6.35 | vacu |
| PeTIP4;1-2 |  | PH02V02Gene26699.t1 |  | 251 | 25.57 | 6 | 6.35 | vacu |
| PeTIP4;2 | PH01001821G0300 | PH02V02Gene10886.t1 | FP098139.1 | 250 | 25.31 | 6 | 6.74 | vacu |
| PeTIP4;2-1 |  | PH02V02Gene42684.t1 |  | 249 | 25.09 | 6 | 6.27 | vacu |
| PeTIP4;3 |  | PH02V02Gene42683.t1 |  | 250 | 25.34 | 6 | 6.63 | vacu |
| PeTIP4;3-1 |  | PH02V02Gene10888.t1 |  | 250 | 25.28 | 6 | 6.63 | vacu |
| PeTIP5;1 |  | PH02V02Gene01979.t1 |  | 262 | 27.13 | 6 | 6.74 | vacu |
| PeTIP5;2 |  | PH02V02Gene24321.t1 |  | 262 | 24.60 | 6 | 7.77 | vacu |
| PeTIP5;3 |  | PH02V02Gene28606.t1 |  | 257 | 26.08 | 6 | 7.05 | vacu |
| PeNIP1;1 | PH01000253G1030 | PH02V02Gene20722.t1 | FP095251.1 | 276 | 29.44 | 6 | 9.24 | plas |
| PeNIP1;2 | PH01002816G0020 | PH02V02Gene32017.t1 |  | 276 | 29.39 | 6 | 9.05 | plas |
| PeNIP1;3 | PH01001769G0230 | PH02V02Gene42153.t2 |  | 280 | 29.58 | 6 | 6.51 | plas |
| PeNIP1;4 | PH01001725G0380 |  |  | 241 | 25.50 | 4 | 7.01 | plas |
| PeNIP1;5 |  | PH02V02Gene42932.t1 |  | 284 | 29.91 | 6 | 8.65 | plas |
| PeNIP1;6 |  | PH02V02Gene08258.t2 |  | 283 | 29.70 | 6 | 8.64 | plas |
| PeNIP1;7 |  | PH02V02Gene21984.t1 |  | 280 | 30.19 | 6 | 8.99 | plas |
| PeNIP2;1 | PH01000135G1100 | PH02V02Gene01049.t2 |  | 295 | 31.79 | 6 | 6.7 | plas |
| PeNIP2;2 | PH01000065G1650 | PH02V02Gene10048.t1 |  | 295 | 31.69 | 6 | 7.69 | plas |
| PeNIP2;3 |  | PH02V02Gene06468.t1 |  | 211 | 22.62 | 5 | 7.09 | plas |
| PeNIP2;4 |  | PH02V02Gene43511.t1 |  | 297 | 31.79 | 6 | 7.1 | plas |
| PeNIP3;1 | PH01001509G0200 |  |  | 320 | 33.04 | 5 | 5.45 | plas |
| PeNIP3;2 | PH01000508G0290 | PH02V02Gene12611.t1 |  | 260 | 27.59 | 5 | 5.87 | plas |
| PeNIP3;3 |  | PH02V02Gene21441.t1 |  | 295 | 30.68 | 6 | 9.08 | plas |
| PeNIP3;3-1 |  | PH02V02Gene10427.t1 |  | 292 | 30.48 | 6 | 8.64 | plas |
| PeNIP3;4 |  | PH02V02Gene12614.t1 |  | 236 | 24.03 | 4 | 7.93 | plas |
| PeNIP3;5 |  | PH02V02Gene12613.t1 |  | 286 | 29.43 | 6 | 7.96 | plas |
| PeSIP1;1 | PH01000897G0530 |  |  | 247 | 26.01 | 6 | 9.25 | plas |
| PeSIP1;1-1 |  | PH02V02Gene19002.t1 |  | 247 | 26.05 | 6 | 9.1 | plas |
| PeSIP1;1-2 |  | PH02V02Gene45856.t1 | FP093349.1 | 248 | 26.18 | 6 | 9.1 | cyto/vacu |
| PeSIP2;1 | PH01000430G0050 | PH02V02Gene18143.t1 |  | 251 | 27.08 | 6 | 9.94 | plas |

plas: plasma membrane; vacu: vacuolar membrane; chlo: chloroplast; cyto: cytosol.

Figure S1: Expression profiles of sugar transport genes in different tissues of moso bamboo. A. Roots formed on rhizomes, and those generated from the base of shoots with 0.1/0.5/2/10 cm length and mature roots with lateral roots; B. Rhizomes; C. Rhizome buds; D. Shoot samples collected from the top/middle/basal portions of shoots at four development stages (0.2, 1.5, 3.0, and 6.7 m, respectively); E. Buds, buds formed on the top/ middle/basal portions of 3.0 m shoots; F: Leaves, including leaf blades and leaf sheaths; G. Sheath sheets. The heat map was established using Matrix2png software. The scale represents the signal intensity of FPKM values after normalized by log_2_.

Figure S2: Diurnal variations in the root pressure of moso bamboo shoots in the second day (7:00 p.m. on 21 April 2017 to 7:00 p.m. on 22 April 2017).

The truncated bamboo shoots were measured, and only half of the bamboo shoots could be used in the second day, which the values of root pressure were much lower than those measured in the first day.

Figure S3: Diurnal variations in the root pressure of moso bamboo shoots measured in another growing season (Pre-experiment).
